# The Impact of Nutrient Intake and Metabolic Wastes during Pregnancy on Offspring Hypertension: Challenges and Future Opportunities

**DOI:** 10.3390/metabo13030418

**Published:** 2023-03-12

**Authors:** You-Lin Tain, Chien-Ning Hsu

**Affiliations:** 1 Division of Pediatric Nephrology, Kaohsiung Chang Gung Memorial Hospital, Kaohsiung 833, Taiwan; 2 College of Medicine, Chang Gung University, Taoyuan 333, Taiwan; 3 Institute for Translational Research in Biomedicine, Kaohsiung Chang Gung Memorial Hospital, Kaohsiung 833, Taiwan; 4 Department of Pharmacy, Kaohsiung Chang Gung Memorial Hospital, Kaohsiung 833, Taiwan; 5 School of Pharmacy, Kaohsiung Medical University, Kaohsiung 807, Taiwan

**Keywords:** hypertension, nutrient-sensing signal, developmental origins of health and disease (DOHaD), asymmetric dimethylarginine, uremic toxin, short chain fatty acid, trimethylamine-N-oxide, AMP-activated protein kinase (AMPK)

## Abstract

Hypertension can have its origin in early life. During pregnancy, many metabolic alterations occur in the mother that have a crucial role in fetal development. In response to maternal insults, fetal programming may occur after metabolic disturbance, resulting in programmed hypertension later in life. Maternal dietary nutrients act as metabolic substrates for various metabolic processes via nutrient-sensing signals. Different nutrient-sensing pathways that detect levels of sugars, amino acids, lipids and energy are integrated during pregnancy, while disturbed nutrient-sensing signals have a role in the developmental programming of hypertension. Metabolism-modulated metabolites and nutrient-sensing signals are promising targets for new drug discovery due to their pathogenic link to hypertension programming. Hence, in this review, we pay particular attention to the maternal nutritional insults and metabolic wastes affecting fetal programming. We then discuss the role of nutrient-sensing signals linking the disturbed metabolism to hypertension programming. This review also summarizes current evidence to give directions for future studies regarding how to prevent hypertension via reprogramming strategies, such as nutritional intervention, targeting nutrient-sensing signals, and reduction of metabolic wastes. Better prevention for hypertension may be possible with the help of novel early-life interventions that target altered metabolism.

## 1. Introduction

Hypertension is nowadays a top risk factor for cardiovascular disease (CVD) [1,2]. Though many antihypertensive drugs and interventions have been developed for hypertension [3], the prevalence of hypertension continues to rise worldwide [4]. All this raises the level of concern for prevention and not just treatment of hypertension.

Human and animal evidence reveals that hypertension could have its origin in prenatal life and in early childhood [5,6]. Nowadays this theory referred to as “developmental programming” or “the developmental origins of health and disease” (DOHaD), which proposes that adaptations to adverse intrauterine environments alter structure and function of organs during fetal development [7,8]. Several maternal conditions occurring during organogenesis can give rise to the development of hypertension during adult life, including nutritional imbalance, illness, pollutant exposure, and medication use [7,8]. Interestingly, these conditions were more or less the same as those factors involved in metabolic disease [9,10].

The major functions of metabolism are: energy production; the conversion of nutrients to carbohydrates, proteins, and lipids; and the elimination of metabolic wastes. Hypertension is connected to impaired metabolic homeostasis [11], although it is not clear whether disturbed metabolism is a cause or a consequence. Hence, understanding of the impact of disturbed metabolism in hypertension programming is important, as targeting metabolic changes might provide novel therapeutic opportunities to avert programmed hypertension.

The aim of our review was to provide insight into how maternal nutritional insults and metabolic wastes impact offspring hypertension, what are the mechanisms behind hypertension programming, and what is our potential strategy for targeting metabolic changes to prevent hypertension.

## 2. Nutrition and Metabolism during Pregnancy and Fetal Development

Maternal nutrition has substantial implications for fetal development. It not only affects the maternal metabolic adjustment capacity to the hormones secreted by the placenta but it is also the only way for the fetus to get the required nutrients. Requirements for quite a lot of nutrients rise during gestation to meet maternal and fetal demands, which require an increased consumption.

During gestation many metabolic alterations occur in the mother that are created for supporting fetal development. For example, the mother becomes less reactive to insulin, resulting in increased glucose availability to the fetus in late gestation. Dietary proteins perform a broad spectrum of metabolic and biological functions. In addition to being the basic building blocks of proteins, amino acids are involved in the regulation of blood pressure (BP), lipid metabolism, food intake and immune function [12]. In early pregnancy, protein turnover is similar to that of non-pregnant women; there is an increase in protein synthesis by 15% in the second trimester and 25% during the third trimester [13].

Hypoaminoacidemia happens in gestation during fasting, especially glucogenic amino acids [13,14]. The amounts of circulating amino acids have been close linked to fetal outcomes, particularly to infant birth weight. Accordingly, the current Dietary Reference Intake is 1.1 g/kg/day of protein during gestation, which is moderately higher than the 0.8 g/kg/day recommended in the non-pregnant state [15]. Failure to adjust the mother’s body to the pregnant state may induce compromised pregnancy and impair fetal development.

Moreover, fetal metabolic wastes are transferred into the maternal circulation via the placenta and eliminated by maternal urination. Accordingly, neonates born to mothers with chronic kidney disease (CKD) are at risk for preterm birth, low birth weight, small size for gestational age, stillbirth and neonatal mortality [16].

### 2.1. Maternal Malnutrition and Offspring Hypertension in Humans

Both undernutrition and overnutrition during pregnancy have increased risk for hypertension in later life. Macronutrients are nutrients that people require in large quantities to generate energy, mainly carbohydrates, proteins and fats. Unlike macronutrients, micronutrients are vitamins and minerals which are consumed in small quantities, but are nonetheless essential for physical function. Emerging human and animal evidence supports the idea that excess or deficits in specific nutrients are related to hypertension of developmental origins.

Table 1 lists a summary of human studies documenting offspring hypertension coincident with nutritional imbalance during pregnancy [17,18,19,20,21,22,23]. First, associations between maternal undernutrition and offspring hypertension are supported by several famine cohort studies [17,18,19,20]. The studies on the Dutch famine of 1944–1945 offer a clear look at how undernutrition in pregnancy is associated with increased risk for developing adverse outcomes in adult offspring. One study recruited a cohort of 2414 people, aged 50 years, born around the time of the 1944–1945 Dutch famine, of whom 741 subjects developed not only hypertension, but also hyperlipidemia, obesity, and insulin resistance. These findings indicate that maternal undernutrition has an important impact on offspring health in later life, but that the timing of the nutritional insult determines which organ system is affected [17,18]. Studies in different famine cohorts suggest maternal undernutrition increases hypertension risk without racial disparities [17,18,19,20]. However, scarce information currently exists with regard to the link between specific nutrient deficiencies in gestation and offspring hypertension [24,25].

Overnutrition refers to a type of malnutrition caused by consuming too much of a certain nutrient, particularly in an imbalanced ratio. Today, only a few human studies have surveyed the impact of excessive intake of a certain macronutrient during gestation on adverse offspring outcomes. Prior work revealed that a high protein diet in gestation is associated with hypertension in adulthood [23,24,25]. A previous study of 253 subjects from Scotland who were born to mothers who had high daily animal protein (>50 g) and low carbohydrate intakes in late pregnancy showed an association with high BP at 40 years of age [21]. Additional studies support the notion that higher maternal dietary protein intake at the expense of carbohydrates is associated with offspring hypertension in adulthood [22,23].

### 2.2. Animal Models of Maternal Malnutrition-Induced Programmed Hypertension

Various animal models have been established using insufficient or excessive intake of a specific nutrient during gestation and/or lactation to validate the associations between maternal malnutrition and offspring hypertension found in human observational studies. Here, we summarize current knowledge on maternal malnutrition-induced offspring hypertension in various rodent models (Table 2) [26,27,28,29,30,31,32,33,34,35,36,37,38,39,40,41,42,43,44,45,46,47,48,49,50,51,52,53,54,55]. Considering that one rat month is comparable to three human years [56], the ages of rats developing hypertension are approximately equivalent to humans from childhood to old age.

There are different methods used for inducing malnutrition in pregnancy and fetus. The most well-established are 2 models of maternal under-nutrition (caloric and protein restriction) and some models of maternal over-nutrition. These maternal over-nutrition models, which are typical of the Western diet (high sugar, fat, and salt), result in a disturbed fetal nutritional environment and metabolism [57]. This in turn leads to a disturbed metabolic profile, such as insulin resistance, obesity, diabetes, and fatty liver, in the adult offspring [9,10].

Caloric restriction refers to a reduction in caloric intake without incurring deprivation of essential nutrients. Similar to prior research on the effects of famine in humans, restricting caloric intake to 30–70% of normal in pregnant rats caused hypertension in their adult offspring [26,27,28,29]. Generally speaking, pups exposed to severe caloric restriction were more likely to have hypertension earlier. Similar to caloric restriction, a protein restriction model has also been commonly utilized to evaluate nutritional programming-induced offspring hypertension [30,31,32,33]. Protein restriction, ranging from 6 to 9%, induced an increase in BP in rat offspring, demonstrating a tendency for those with severe protein restrictions to show earlier development of hypertension [30,31,32,33]. Additionally, a deficiency of specific amino acids, such as methionine [34] and tryptophan [35], in gestation and lactation has also been reported regarding programmed hypertension. Furthermore, deficiencies in other nutrients, including salt [36], calcium [37], iron [38], vitamin D [39], zinc [40], folic acid and vitamins B2, B6, and B12 [34] in pregnant rats were also related to hypertension in their adult progeny.

On the other hand, over-nutrition arising from excessive intake of specific nutrients can lead to hypertension programming [58]. Feeding pregnant rats a diet high in sucrose or fructose induced hypertension in their offspring [41,42,43,44,45,46]. A maternal high-fructose diet not only caused hypertension, but also obesity, insulin resistance, and fatty liver [59]. A high-fat diet is a broadly used model for studying metabolic disease of both established and developmental origins [60,61]. Despite a high-fat diet altering fetal programming and resulting in offspring hypertension, the programming effects may vary depending on age, sex, strains, and different compositions of fats [62]. Notably, in animal models of maternal diets characterized by high-sugar drinks, high-fat products, and excess salt characteristic of the human Western diet, synergistic effects of these key components on the rise of BP in adult progeny were detected [42,48,49,54]. Moreover, male rat offspring exposed to excessive protein [56], methyl-donor [34], or salt [36] in maternal intake were also characterized by raised BP.

Worthy of note is that hypertension programming can be induced by maternal malnutrition and disturbed metabolism in a variety of animal models. No matter what type of nutritional imbalance, they produce the same end result–hypertension. These observations reveal there might be common mechanisms underlying hypertension programmed by maternal nutritional insults.

### 2.3. Impact of Metabolic Wastes in Pregnancy on Programmed Hypertension

The biochemical parameters of waste products in the form of blood creatinine and urea demonstrated a significant drop from the pre-pregnancy values by 24 weeks of pregnancy, but the values later rise in the third trimester in contradistinction to the basically expected trends [63]. The excretion of waste products is closely dependent of renal function. Accordingly, chronic kidney disease (CKD) in pregnancy causes accumulation of more waste products, which can be detrimental to the fetal development [63].

The prevalence of CKD in women of childbearing age is around 3%–4% [64]. However, too little attention has been focused on the identification of hypertension in children born to mothers with CKD. Using animal models, maternal uremia-induced adverse offspring outcomes have been evaluated in an adenine-induced maternal CKD model [65]. In this model, maternal CKD-primed offspring hypertension is associated with increased uremic toxin asymmetric dimethylarginine (ADMA), increased trimethylamine N-oxide (TMAO), and reduced microbiota-derived metabolite acetate and butyrate levels [66,67]. These findings suggest a pathogenic link between excessive metabolic wastes in pregnancy and the development of hypertension later in life.

## 3. The Link between Nutrient-Sensing Signals, Disturbed Metabolism, and Programmed Hypertension

**Figure 1:**
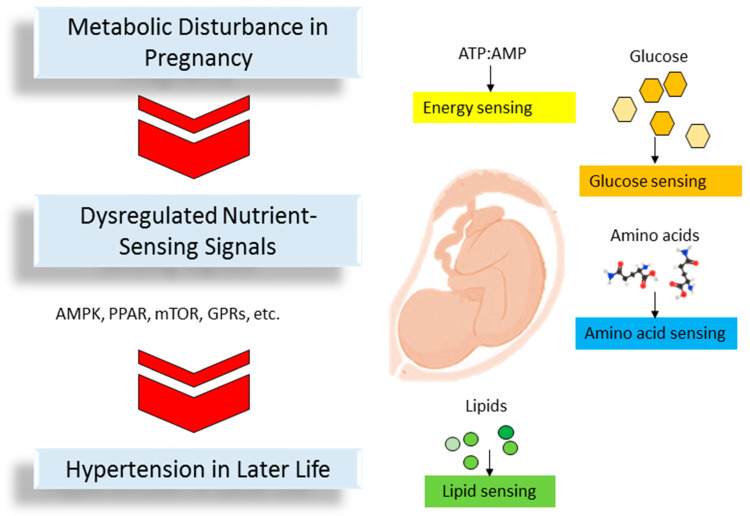
Schematic diagram highlighting the various nutrient-sensing signals in pregnancy that may impact fetal programming resulting in hypertension in later life. These signals cover AMP-activated protein kinase (AMPK), peroxisome-proliferator activated receptors (PPARs), the mechanistic target of rapamycin (mTOR), and G-coupled protein receptors (GPRs).

Mammalian cells have various ways of sensing energy and essential cellular nutrients such as amino acids, glucose, and lipids. The sensing of nutrient signals is the key factor for whole-body metabolic homeostasis [68]. Maternal dietary nutrients act as metabolic substrates for various metabolic processes via nutrient-sensing signals. During pregnancy, nutrient-sensing mechanisms can detect the range of specific nutrients to ensure that fetal growth rate and organic function coordinate properly. Conversely, disturbed nutrient-sensing signals in pregnancy result in adverse fetal programming and have a pathogenic role in the developmental programming of hypertension [69,70]. Here we summarize current evidence documenting how these nutrient-sensing signals become deregulated in programmed hypertension and describe the underlying mechanisms (Figure 1).

### 3.1. Energy Sensing

To ensure cellular metabolism, the ATP level must be tightly regulated within a proper range. AMP-activated protein kinase (AMPK) appears to achieve this coordination [71]. AMPK is a phylogenetically conserved, ubiquitously expressed serine/threonine protein kinase containing catalytic α subunits and regulatory β and γ subunits [71]. AMPK is known to be activated by falling cellular energy status, signaled by increasing AMP-to-ATP or ADP-to-ATP ratios and acts to restore energy homeostasis by stimulating energy production.

Emerging evidence suggests that dysregulated AMPK signaling pathway is connected to developmental programming of hypertension, whereas AMPK activation in early life helps prevent offspring’s hypertension [72]. In spontaneously hypertensive rat (SHR), aortic AMPK activity was reduced, whilst 5-aminoimidazole-4-carboxamideriboside (AICAR), an AMPK activator, can reduce BP in SHRs [73]. Similarly, other AMPK activators, such as metformin and resveratrol can block the development of hypertension in SHRs [74,75]. In a perinatal high-fat diet model, resveratrol protected against hypertension coinciding with increased protein level of phosphorylated AMPK2α in offspring kidneys [76]. Likewise, metformin or AICAR protected adult offspring against perinatal high-fat diet-induced hypertension [77,78]. Based on these observations, dysregulated energy sensing is involving in programmed hypertension and interventions that activate AMPK might be expected to be useful in the prevention of hypertension.

### 3.2. Glucose Sensing

Mammals depend on several approaches to maintain glucose concentrations within a narrow physiological range. Multiple mechanisms of glucose sensing exist to tightly regulate the intake, storage and breakdown of glucose by different organs. Additionally, a network of hormone signals, exemplified by glucagon and insulin, seek to coordinate orderly responses to systemic glucose concentrations in distant organs. AMPK is known to participate in controlling glycogen and glucose metabolism.

Unlike glucose, fructose is specifically and passively transported by the facilitative glucose transporter 5. So far, the role of glucose transporters in hypertension remains largely unknown. Despite the similarity in their structures, fructose and glucose are metabolized in different ways. Using the maternal high-fructose diet model, we utilized RNA next-generation sequencing (NGS) technology to investigate the transcriptome expression in several organs [79,80,81]. We found that maternal high-fructose diet caused long-term transcriptome changes. Especially, offspring hypertension coincided with several differentially expressed genes (DEGs) related to fatty acid metabolism, fructose metabolism, insulin signaling, and glycolysis/gluconeogenesis in neonate offspring’s kidneys [81].

### 3.3. Amino Acid Sensing

Amino acids are the building blocks for proteins. Placental amino acid transporters regulate their exchange from the maternal to the fetal circulation [82,83]. As reviewed elsewhere, three principal transport systems account for amino acid uptake in the placenta: exchange, accumulative, and facilitated transporters [84]. Increased renal expression/activity of the solute carrier (SLC) 7A5 and SLC7A8 [85] and decreased expression of SLC7A1 [86] have been related to hypertension. However, the role of placental amino acid transporters in hypertension programming has not been fully established.

The availability of amino acids is highly dependent on the mechanistic target of rapamycin (mTOR) [87]. mTOR functions via two multiprotein complexes termed mTOR complex 1 (mTORC1) and 2 (mTORC2) [88]. Prior research demonstrated that activities of placental mTOR and amino acid transporters were reduced in intrauterine growth retardation (IUGR) [89]. Considering IUGR is a risk factor for developing adulthood hypertension [90], it is very likely that amino acid sensing and mTOR are involved in the mechanisms behind programmed hypertension, although how this integration occurs awaits clarification. In a combined high-fructose and high-salt diet model, the beneficial actions by which maternal melatonin therapy protects adult rat offspring against hypertension were associated with increased renal protein level of mTOR [49].

### 3.4. Lipid Sensing

Lipid metabolism refers to activities from uptake of lipids in the gut to cellular uptake and transport to compartments such as mitochondria. Phosphoinositides are lipid signaling molecules that act as master regulators of cellular signaling. The phosphoinositide signaling system is common to many vasoconstrictor agents and as such is influential in the regulation of BP [91]. However, no information exists regarding their impact on programmed hypertension. AMPK also contributes to lipid metabolism through reduction of fatty acid synthesis and thus inhibition of lipogenesis.

Several nuclear hormone receptors are lipid-sensing factors that influence lipid metabolism [92]. The liver X receptors (LXRs) and peroxisome-proliferator activated receptors (PPARs), working together with PPARγ coactivator-1α (PGC-1α), have been shown to regulate lipid metabolism. AMPK can phosphorylate PGC-1α [93], to mediate the expression of PPAR target genes. mTOR has also been shown to regulate PPAR activation [94]. We previously revealed that several PPAR target genes are involved in hypertension programming, such as *Sod2*, *Sirt7*, *Ren*, *Nrf2*, *Nos2*, *Nos3* and *Sgk1* [95]. Additionally, our prior work reported that the PPAR signaling pathway is involved in animal models of hypertension programming, such as maternal caloric restriction [96] and maternal high-fructose diet [81]. Considering the crucial role of PPARs in the pathogenesis of hypertension, disturbed lipid sensing in response to maternal nutritional insults is likely to have close link to hypertension programming.

The sensing of free fatty acids is through G-coupled protein receptors (GPRs), also referred as free fatty acid receptors (FFARs) [97]. Short chain fatty acids (SCFAs) are the key microbial metabolites formed during bacterial fermentation of dietary fibers, mainly including acetate, butyrate, and propionate [97]. SCFAs are able to activate GPR41 and GPR43, while long chain fatty acids have the ability to activate GPR40 and GPR120. During pregnancy, SCFAs and their receptors have been reported to determine the development and metabolic programming of the fetus [98].

In a maternal high fructose diet model, the elevation of offspring’s BP was accompanying by decreased renal GPR41 and GPR43 expression [45]. Another study demonstrated that perinatal 2,3,7,8-tetrachlorodibenzo-p-dioxin (TCDD) exposure-primed hypertension in adult offspring coincided with downregulation of renal GPR43 expression [99]. These results support the notion that lipid sensing might be a decisive mechanism underlying hypertension programming.

It is well-known that supplementation with long-chain omega-3 polyunsaturated fatty acids (PUFAs) is associated with reduced cardiovascular risk [100]. PUFAs function not only by activating GPR40 and GPR120, but possess also effects on PPARs and other nuclear receptors [100]. Recent evidence suggests a maternal diet rich in PUFAs in pregnancy may reduce the child’s aortic stiffness, with potential benefits of prevention for later CVD [101,102]. As PUFAs have been used as dietary supplements with various health claims, there is a growing need to better realize the lipid sensing signals of action of PUFAs, and to be able to explore mechanisms underlying hypertension.

### 3.5. Other Common Mechanisms

In addition to nutrient-sensing signals, several molecular mechanisms involved in hypertension programming have been proposed based on prior research, covering oxidative stress [65], nitric oxide (NO) deficiency [103], aberrant renin-angiotensin system (RAS) [104], epigenetic regulation [105], increased sympathetic nerve activity [106], sex differences [107], gut microbiota dysbiosis [108], and impaired sodium transport [109]. Some of them are interrelated to nutrient-sensing signals in response to disturbed metabolism in pregnancy, contributing to the development of hypertension. For the sake of brevity, brief references are given below. First, extensive experimental animal studies have revealed the interconnections among NO, oxidative stress, and nutrient-sensing signals involved in hypertension programming [65]. A second line of evidence comes from the balance between AMPK and the RAS [110]. Previous research indicated that AMPK activator can inhibit the classic RAS axis while enhancing the non-classic RAS axis to modulate the RAS balance in favor of vasodilatation. Third, nutrition influences epigenetic processes on multiple levels. Nutrients either directly mediate the production of epigenetic enzymes (i.e., histone deacetylase inhibitors), or alter the substrate availability for enzymatic reaction, thus impacting hypertension-related gene expression [105,111]. Last, recent evidence revealed that the gut microbiota can impact the gut-brain axis controlling energy balance and the gut-kidney axis regulating BP, at both the level of gut nutrient-sensing mechanisms and other organ systems [112,113].

**Figure 2:**
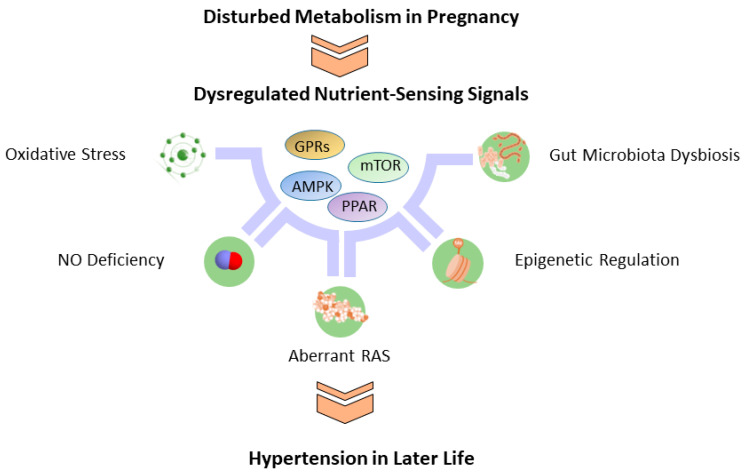
Illustration of dysregulated nutrient-sensing signal interconnected with other molecular mechanisms related to developmental programming of hypertension. AMPK = AMP-activated protein kinase (AMPK); PPAR = peroxisome-proliferator activated receptor; mTOR = the mechanistic target of rapamycin (mTOR); GPRs = G-coupled protein receptors; NO = nitric oxide; RAS = renin-angiotensin system.

As detailed descriptions of these mechanisms are beyond the scope of this paper, readers are referred elsewhere for more in-depth information. A schematic summarizing the dysregulated nutrient-sensing signal and its interconnected molecular mechanisms linked to developmental programming of hypertension is presented in Figure 2.

## 4. Reprogramming Strategy

Emerging evidence reveals a highly disturbed metabolism in different pathways in response to multiple maternal insults, resulting in programmed hypertension. These data point to new potentially causal mechanisms behind metabolic programming, which can be further investigated in the search for new ways of preventing hypertension.

**Figure 3:**
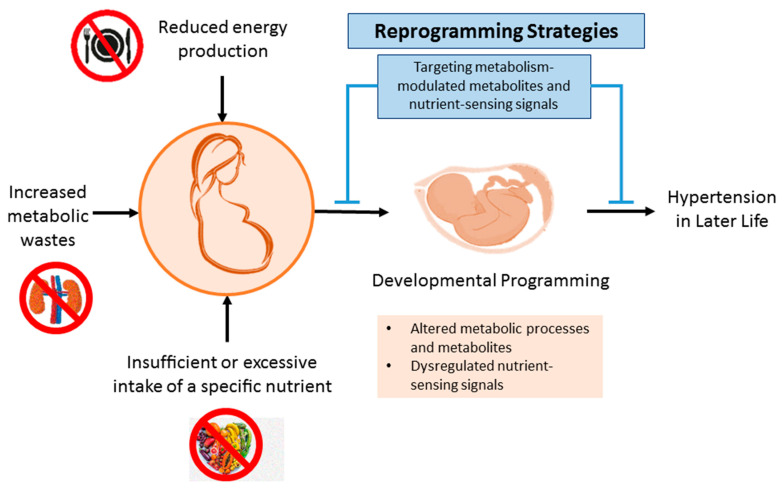
Schematic representation of the interrelationships between disturbed metabolism in pregnancy, developmental programming of hypertension, and reprogramming strategies.

As for our current understanding of the DOHaD theory, it turns out that control and prevention of hypertension can be initiated early before the onset of hypertension in early life stage-fetal periods, namely reprogramming [114]. Particularly, the mechanisms involved in disturbed metabolism-related hypertension programming mentioned above may serve as potential targets for reprogramming. Toward this end, interventions to offset programming processes behind hypertension that have been assessed can be categorized as three types: nutritional intervention, targeting nutrient-sensing signal, and reduction of metabolic wastes. The interrelationships between maternal metabolic disturbance, developmental programming of hypertension, and reprogramming strategies are illustrated in Figure 3.

### 4.1. Nutritional Intervention

It is well known that optimal maternal nutrition is essential for supporting fetal growth and development. Accordingly, the above-mentioned risk factors regarding maternal malnutrition illustrated in Table 1 should be avoided during pregnancy and lactation. In addition, perinatal supplementation with certain nutrients can be beneficial in relation to offspring hypertension programmed by various early-life insults as we reviewed elsewhere [115]. These nutritional interventions cover macronutrients (protein, lipid, and carbohydrate) and micronutrients (folic acid, vitamin C, E, and selenium) (Table 3). Macronutrients already being utilized in reprogramming strategies are largely amino acids. Little reliable data presently exists about the reprogramming effects of specific micronutrients on hypertension programming.

#### 4.1.1. Protein

Nutritional supplementation interventions starting during gestation as a reprogramming intervention to avert developmental programming of hypertension in rodent models are listed in Table 3 [27,28,35,45,55,116,117,118,119,120,121,122,123,124,125,126,127,128,129,130]. Among them, amino acids are the most commonly used nutrients for prevention of programmed hypertension. Vasoactive properties of dietary proteins depend on the amino acid compositions [102], which can regulate BP homeostasis. For example, serine, taurine, alanine, and glycine produce depressor responses, while proline, glutamate, aspartic acid, and asparagine yield pressor responses in conscious rats [131].

First, taurine has several potentially beneficial effects against hypertension that include the regulation of NO, oxidative stress, and the RAS [132]. Previous studies showed that dietary taurine supplementation from gestation to lactation can prevent offspring’s hypertension programmed by a maternal high-sugar diet [116] or streptozotocin (STZ)-induced maternal diabetes [117]. Citrulline is a non-essential amino acid and is available as a dietary supplement [133]. As citrulline can be transformed to arginine for NO production, oral citrulline treatment has been considered as an add-on therapy to enhance NO synthesis [133]. Additionally, previous research demonstrated that citrulline supplementation can act as a reprogramming intervention in several rat models of programmed hypertension, including maternal caloric restriction [28], antenatal dexamethasone administration [118], maternal STZ-induced diabetes [119], and maternal N^G^-nitro–L-arginine methyl ester (L-NAME) administration [120]. Moreover, other amino acids, like glycine, cysteine, tryptophan, and branched-chain amino acid have also shown beneficial effects on programmed hypertension [121,122,123,124]. Cysteine is a sulfur-containing amino acid and acts as a component of glutathione. Formerly we reported that rat offspring born to dams with CKD supplemented with D- or L-cysteine during gestation were protected against hypertension at 12 weeks of age [122]. As cysteine is the substrate for hydrogen sulfide (H_2_S), early-life cysteine supplementation is a way to produce endogenous H_2_S and prevent the developmental programming of hypertension [134].

Although current hypertension guidelines recommend the adoption of dietary modifications in patients with elevated BP [135], whether a Mediterranean diet in pregnant women can help prevent offspring hypertension has yet to be studied. A high intake of animal protein, particularly red meat, which contains high levels of methionine, is related to vascular ageing and CVD [136]. In contrast, a Mediterranean diet, characterized by higher plant-based foods and lower red meat intake, is related to lower CVD risk. Considering the vasoprotective functions of a Mediterranean diet [137], further study is necessary to elucidate whether specific restriction levels of animal protein or individual amino acids (e.g., methionine) may serve as reprogramming interventions for hypertension.

During pregnancy and lactation, milk and dairy products consumption is a major source of source of protein and other nutrients. A systematic review of 20 studies indicated that maternal milk and dairy products intake during pregnancy is positively associated with fetal outcome [138]. Due to their complex biochemistry, the association between milk/dairy consumption and CVD and all-cause mortality remains inconclusive [139]. So far, the lack of studies prevents any conclusions being drawn related to hypertension programming.

#### 4.1.2. Lipids

Several types of lipids have been utilized as reprogramming interventions for programmed hypertension in experimental studies. Conjugated linoleic acid is a microbial metabolite coming from dietary PUFAs. Conjugated linoleic acid supplementation in gestation and lactation protected adult rat offspring against high-fat diet-primed offspring hypertension [125]. Another report demonstrated that maternal omega-3 PUFAs supplementation has reprogramming effects against offspring hypertension programmed by maternal low protein intake [126]. So far, four reports indicated reprogramming effects of SCFA supplementation on programmed hypertension. Acetate, butyrate, and propionate have been utilized as reprogramming interventions in models of maternal high-fructose diet [127], maternal minocycline exposure [128], maternal tryptophan-free diet [35], and maternal CKD [129], respectively.

#### 4.1.3. Carbohydrate

There are some carbohydrates being utilized to prevent offspring’s hypertension as reprogramming interventions. Some known prebiotics (inulin or oligosaccharides) are low digestible carbohydrates [140]. Perinatal long chain inulin supplementation was able to protect adult rat progeny against hypertension programmed by maternal high-fructose or high-fat diet [45,55].

#### 4.1.4. Micronutrients

Several vitamins and trace elements show cardiovascular benefits [141]. Unlike macronutrients, only two studies reported the impact of maternal micronutrients supplementation on offspring hypertension [27,130]. One previous report demonstrated that gestational supplementation with folic acid, vitamin C, E, and selenium averted maternal caloric restriction-induced hypertension [27]. Considering these micronutrients have antioxidant properties, their reprogramming effects on programmed hypertension is presumably accompanied by reduction of oxidative stress [58]. Folic acid, a key player in one-carbon metabolism, also showed benefits in preventing hypertension programmed by maternal low protein diet [130].

Dietary sodium and potassium intake is also involved in the regulation of BP. Prior work revealed that a diet that reduces salt intake while enhancing potassium consumption is able to control or prevent hypertension [142]. Nevertheless, a previous study reported that both high and low maternal salt intake in pregnancy resulted in offspring hypertension [36]. Thus, there remains a lack of evidence supporting sodium reduction and increased potassium intake for prevention of hypertension programming.

#### 4.1.5. Others

There are several functional foods that can improve hypertension as well as metabolic abnormalities [143,144]. For example, chocolate or cocoa product consumption significantly improved vascular function in a human trial [145]. Although metabolic derangements in rat offspring born to chocolate fed dams have been reported [146], whether chocolate supplementation in pregnancy can avert hypertension in offspring is still unclear.

Additionally, plants produce polyphenols, which are considered essential functional foods in our diet. As we reviewed elsewhere [147], several polyphenols, including stilbenes [47,75], tannins [50], flavonols [70], and flavanols [148], have shown benefits against offspring’s hypertension programmed by various maternal insults evaluated in animal models.

### 4.2. Targeting Nutrient-Sensing Signal

Malnutrition and disturbed metabolism in early life can impair nutrient-sensing signals that have a fundamental impact on fetal metabolism and development. Interventions targeting AMPK or PPARs signaling have been reported to avert the development of hypertension in a variety of developmental programming models.

#### 4.2.1. AMPK

Both indirect and direct AMPK activators have been examined in developmental programming of hypertension. Indirect AMPK activators refers to modulators that cause AMP or calcium accumulation without a direct communication with AMPK [149]. Some indirect AMPK activators have shown their benefits on programmed hypertension, including resveratrol [47,76], metformin [77], quercetin [148], epigallocatechin gallate [150], and garlic [151]. Besides, the use of direct AMPK pan-activator AICAR in gestation and lactation can protect adult progeny against hypertension programmed by a high-fat diet [78]. Nevertheless, contemporary knowledge of isoform-specific AMPK activators in the developmental programming of hypertension is significantly limited.

Of note, resveratrol recently received great attention as a reprogramming intervention not only against hypertension but also metabolic syndrome [152,153,154]. In a combined high-fat diet and L-NAME administration model, resveratrol therapy protected adult offspring against hypertension related to activation of the AMPK/PGC1α pathway [155]. In addition, resveratrol has the ability to prevent the elevation of offspring’s BP via AMPK activation in a high-fructose diet model [47] and a high-fat diet model [76]. These observations support the idea that the interaction between resveratrol and nutrient-sensing signals are implicated in hypertension of developmental origins.

#### 4.2.2. PPAR

A growing body of evidence indicated that PPARs have a key role in the pathogenesis of many metabolic disorders, and their ligands have therapeutic potential in restoring these metabolic disorders [95,156,157]. Nevertheless, not many studies have evaluated the impact of PPAR modulators on metabolic programming, especially hypertension [95]. Selective PPARγ agonists, pioglitazone and rosiglitazone, can be protective in low protein diet-induced hypertension and genetic hypertension [158,159]. Additionally, some natural PPAR agonists, such as conjugated linoleic acid and omega-3 PUFAs, have been examined in hypertension programming [125,126]. Given that fatty acid derivatives have a widespread range of affinity to PPARs [160], it is hard to determine whether their reprogramming effects on BP are PPAR-dependent or not. Currently, no information exists with regard to the reprogramming effect of PPARβ/δ on programmed hypertension, despite PPARγ modulators having been considered attractive drug targets for addressing metabolic disorders [161].

### 4.3. Reduction of Metabolic Wastes

Uremic toxins are metabolic wastes that accumulate in subjects with impaired kidney function. The major uremic toxins contributing to hypertension programming are TMAO, ADMA, and tryptophan-derived metabolites [162,163,164]. One major mechanism linking uremic toxins to developmental programming of hypertension is gut microbiota dysbiosis [108]. Microbiota dysbiosis, associated with a uremic milieu, is characterized by loss of diversity, shifts in key taxa, reductions in beneficial microbes, and alterations of microbial metabolites, and is involved in the pathogenesis of hypertension [113,165]. Particularly, a connection between microbiota-derived metabolites and offspring hypertension has been found in several developmental programming animal models [45,68,127]. These metabolites include SCFAs, TMAO, and tryptophan-derived metabolites. Importantly, gut microbiota dysbiosis is interconnected with a number of mechanisms behind hypertension programming, such as oxidative stress, inflammation, aberrant RAS, and dysregulated nutrient-sensing signals [108]. Accordingly, gut microbiota-targeted therapy has emerged as a reprogramming strategy to prevent hypertension programmed by numerous maternal insult stimuli [108]. Here, we highlight pathogenic mechanisms behind hypertension programming and the way in which reduction of uremic toxins contributes to prevention of hypertension.

#### 4.3.1. TMAO

TMAO is a gut microbiota-derived uremic toxin and its level correlates with CVD mortality [166]. TMAO production results from the fermentation by the gut microbiota of dietary carnitine and choline, which are transformed to trimethylamine (TMA) and converted into TMAO by flavin-containing monooxygenases in the liver [167]. Two microbial choline TMA lyase inhibitors, iodomethylcholine (IMC) and 3,3-dimethyl-1-butanol (DMB), have been utilized to inhibit TMAO production [168]. Maternal TMAO administration can cause an increase in offspring’s BP [169]. Conversely, DMB or IMC therapy from gestation to lactation averted adult rat progeny against hypertension programmed by various maternal insults, which was related to the restoration of the TMAO metabolic pathway [69,99,127]. These observations theoretically indicated that targeted TMAO reduction may have some potential to avert hypertension, though these results require further clinical translation.

#### 4.3.2. ADMA

Another uremic toxin is ADMA. ADMA is increasingly recognized as a biomarker of CKD and hypertension [170,171]. We and others previously reviewed a lot of currently used drugs which can lower ADMA levels and restore NO bioavailability [170,171]. These ADMA-lowering agents cover telmisartan, melatonin, resveratrol, N-acetylcysteine, atorvastatin, vitamin E, salvianolic acid A, oxymatrine, metformin, rosuvastatin, aliskiren, etc. However, only a few of them have been evaluated in animal models to avert offspring’s hypertension.

Maternal treatment with melatonin [49], aliskiren [96], resveratrol [172], or N-acetylcysteine [173] has been reported to protect adult offspring against programmed hypertension coinciding with reduction of plasma ADMA. However, specific ADMA-lowering agents are still unreachable in clinical practice. Considering that ADMA is metabolized by dimethylaminohydrolase (DDAH)-1 and -2, and that methyltransferase isoenzymes (PRMTs) are responsible for ADMA generation, the discovery of specific DDAHs agonists and PRMT inhibitors should bring advanced therapies to reduce ADMA and restore NO, and thus avert the development of hypertension.

#### 4.3.3. Tryptophan Metabolites

Tryptophan-derived uremic toxins, mostly derived from the kynurenine and indole pathways, have been closely linked to cardiovascular risk in patients with CKD [174]. Indoxyl sulfate and indoleacetic acid are extensively studied uremic toxins. These microbial metabolites derived from tryptophan are potent aryl hydrocarbon receptor (AHR) ligands, by which they exhibit pro-oxidant, pro-inflammatory, and pro-apoptotic properties. It is known that activation of AHR is involved in the pathogenesis of hypertension [175]. AST-120 is an orally administered intestinal sorbent that can adsorb small organic molecules [176]. In CKD, AST-120 could reduce uremic toxins [177,178], while its reprogramming effects on programmed hypertension have not been explored yet.

Considering that tryptophan-derived uremic toxin are ligands for AHR and that AHR activation is associated with hypertension programming [172,179], AHR antagonists might provide a potential reprogramming strategy to prevent tryptophan metabolites-induced adverse outcomes. As a natural AHR antagonist [179], resveratrol has been shown to prevent hypertension programming [153]. Given that resveratrol has multiple biofunctions not just as an AHR antagonist, further research is needed to elucidate whether the use of a specific AHR antagonist can avert offspring hypertension attributed to tryptophan-derived uremic toxins in the future.

## 5. Conclusions and Perspectives

Current evidence has indicated the impact of maternal metabolic disturbance in the developmental programming of hypertension. This review sought to highlight potentially causal mechanisms behind underlying disturbance of metabolism during fetal development and adulthood hypertension. Reflecting current knowledge, our review further opens new avenues for prevention of hypertension via targeting disturbed metabolism underlying hypertension programming.

While prior work has generated ample evidence on the impact of disturbed metabolism during fetal development on the development of adulthood hypertension, it is still uncertain whether and why restoration of metabolic imbalance occurring early in life would benefit offspring outcomes, especially hypertension.

In the future, we recommend bridging the gap between human and animal research through a focus on reprogramming strategies targeting metabolism-modulated metabolites and nutrient-sensing signals. There is presently scant information on how various reprogramming strategies obtained from animal research might be used in pregnant women. Longitudinal analysis of metabolites, nutrient-sensing signals-related biomarkers and detailed background data are of the greatest importance in studying the time window effects of maternal metabolic disturbance on offspring hypertension; this research can aid in design of hypothesis-driven interventions and ideal timing of their administration.

These are imperative questions to answer, considering early-life preventative interventions targeting restoration of metabolic disturbance might provide novel therapeutic opportunities to reduce the global burden of hypertension.

## Figures and Tables

**Table 1:** Summary of Maternal Malnutrition on Offspring Hypertension in Human Studies.

Maternal Malnutrition	Cohort Study/Country	Age at Measure (Year)	Case Number	References
Undernutrition	Dutch famine study/Netherlands	50	741	Painter et al., 2005 [17]
Undernutrition	Dutch famine study/Netherlands	59	359	Stein et al., 2006 [18]
Undernutrition	Biafran famine study/Nigeria	40	1339	Hult et al., 2010 [19]
Undernutrition	China great leap forward famine study/China	55	1029	Li et al., 2017 [20]
High-protein, low-carbohydrate diet	Aberdeen maternity hospital study/Scotland	40	253	Campbell et al., 1996 [21]
High-protein, low-carbohydrate diet	Motherwell study/Scotland	30	626	Shiell et al., 2001 [22]
High-protein intake	DaFO88/Denmark	20	434	Hrolfsdottir et al., 2017 [23]

DaFO88 = Danish fetal origins cohort.

**Table 2:** Summary of Maternal Malnutrition-induced Hypertension Programming in Animal Models.

Animal Models	Intervention Periods	Age at Measure	Species/Gender	References
Under-nutrition				
Caloric restriction, 30%	Gestation	54 weeks	Wistar rat/M+F	[26]
Caloric restriction, 50%	Gestation	16 weeks	Wistar rat/M+F	[27]
Caloric restriction, 50%	Gestation and lactation	12 weeks	SD rat/M	[28]
Caloric restriction, 70%	Gestation days 0–18	28 weeks	Wistar rat/M+F	[29]
Protein restriction, 6%	Gestation	52 weeks	SD rat/F	[30]
Protein restriction, 8.5%	Gestation	20 weeks	SD rat/M	[31]
Protein restriction, 9%	Gestation	12 weeks	Wistar rat/M	[32]
Protein restriction, 9%	Gestation	22 weeks	Wistar rat/M+F	[33]
Methyl-deficient diet	Gestation and lactation	12 weeks	SD rat/M	[34]
Tryptophan-free diet	Gestation and lactation	16 weeks	SD rat/M	[35]
Low-salt diet, 0.07%	Gestation and lactation	21 weeks	SD rat/M	[36]
Calcium-deficient die	Gestation	52 weeks	Wistar-Kyoto rat/M+F	[37]
Iron restriction	4 weeks before conception and throughout pregnancy	3 months	Wistar rat/M+F	[38]
Vitamin D restriction	6 weeks before conception and throughout pregnancy and lactation	8 weeks	SD rat/M+F	[39]
Zinc-deficient diet	Gestation and lactation	12 weeks	Wistar rat/M	[40]
Over-nutrition				
High-sucrose solution, 20%	Gestation	22 months	SD rat/M	[41]
High fructose/salt solution, 10%/4%	4 weeks before conception and throughout pregnancy and lactation	9 weeks	SD rat/M	[42]
High-fructose solution, 20%	Gestation and lactation	8 months	C57BL6J mice/M+F	[43]
High-fructose diet, 60%	Gestation and lactation	12 weeks	SD rat/M	[44,45,46]
Maternal and post-weaning high-fructose diet	Gestation and lactation	12 weeks	SD rat/M	[47]
Maternal high-fructose diet plus post-weaning high-fat diet	Gestation and lactation	12 weeks	SD rat/M	[48]
Maternal high-fructose diet plus post-weaning high-salt diet	Gestation and lactation	12 weeks	SD rat/M	[49]
High-fat diet, 24%	Lactation	22 weeks	Wistar rat/M	[50]
High-fat diet, 25.7%	Lactation	22 weeks	SD rat/F	[51]
High-fat diet, 45%	Gestation and lactation	30 weeks	C57BL6J mice/M	[52]
High-fat diet, 58%	5 weeks before the delivery and throughout pregnancy and lactation	16 weeks	SD rat/M	[53]
High fat plus high-salt diet, 45%/4%	3 weeks before conception and throughout pregnancy and lactation	19 weeks	SD rat/M	[54]
Maternal and post-weaning high-fat diet, 58%	Gestation and lactation	16 weeks	SD rat/M	[55]
High-protein diet	Gestation and lactation	22 weeks	Wistar rat/M	[56]
High methyl-donor diet	Gestation and lactation	12 weeks	SD rat/M	[34]
High-salt diet, 4%	Gestation and lactation	21 weeks	SD rat/M	[36]

Studies tabulated according to types of malnutrition. SD = Sprague-Dawley rat; M = Male; F = Female.

**Table 3:** Nutritional supplementation used as reprogramming interventions to prevent the developmental programming of hypertension in rodent models.

Intervention	Periods	Animal Models	Age at Measure	Species/Gender	References
Protein					
3% taurine	Gestation and lactation	Maternal high-sugar diet	8 weeks	SD rat/F	[116]
3% taurine	Gestation and lactation	Streptozotocin-induced diabetes	16 weeks	Wistar rat/M+F	[117]
0.25% citrulline	Gestation and lactation	Maternal caloric restriction	12 weeks	SD rat/M	[28]
0.25% citrulline	Gestation and lactation	Prenatal dexamethasone administration	12 weeks	SD rat/M	[118]
0.25% citrulline	Gestation and lactation	Streptozotocin-induced diabetes	12 weeks	SD rat/M	[119]
0.25% citrulline	Gestation and lactation	Maternal L-NAME administration	12 weeks	SD rat/M	[120]
3% glycine	Gestation and lactation	Maternal low protein diet	4 weeks	Wistar rat/F	[121]
Oral gavage of D- or L-cysteine 8 mmol/kg/day	Gestation	Maternal CKD	12 weeks	SD rat/M	[122]
Oral gavage of tryptophan 200 mg/kg/day	Gestation and lactation	Maternal CKD	12 weeks	SD rat/M	[123]
BCAA-supplemented diet	Gestation	Maternal caloric restriction	16 weeks	SD rat/M	[124]
Lipid					
Conjugated linoleic acid	Gestation and lactation	Maternal high-fat diet	18 weeks	SD rat/M	[125]
Omega-3 polyunsaturated fatty acids	Gestation and lactation	Maternal low protein diet	6 months	Wistar rat/M+F	[126]
Magnesium acetate 200 mmol/L	Gestation and lactation	Maternal high-fructose diet	12 weeks	SD rat/M	[127]
Magnesium acetate 200 mmol/L	Gestation and lactation	Maternal minocycline exposure	12 weeks	SD rat/M	[128]
Sodium butyrate 400 mmol/L	Gestation and lactation	Maternal tryptophan-free diet	12 weeks	SD rat/M	[35]
Propionate 200 mmol/L	Gestation and lactation	Maternal CKD	12 weeks	SD rat/M	[129]
Carbohydrate					
5% *w/w* long chain inulin	Gestation and lactation	Maternal high-fructose diet	12 weeks	SD rat/M	[45]
5% *w/w* long chain inulin	Gestation and lactation	Maternal high-fat diet	16 weeks	SD rat/M	[55]
Micronutrients					
Folic acid, vitamin C, E, and selenium	Gestation	Maternal caloric restriction	16 weeks	Wistar rat/M+F	[27]
Folic acid 5 mg/kg/day	Gestation	Maternal low protein diet	15 weeks	Wistar rat/M	[130]

## Data Availability

Data are contained within the article.
